# Global research trends at the intersection of coronavirus disease 2019 (COVID-19) and traditional, integrative, and complementary and alternative medicine: a bibliometric analysis

**DOI:** 10.1186/s12906-020-03151-8

**Published:** 2020-11-23

**Authors:** Jeremy Y. Ng

**Affiliations:** Department of Health Research Methods, Evidence, and Impact, Faculty of Health Sciences, McMaster University, Michael G. DeGroote Centre for Learning and Discovery, Room 2112, 1280 Main Street West, Hamilton, ON L8S 4K1 Canada

**Keywords:** Bibliometric analysis, Complementary and alternative medicine, Coronavirus, COVID-19, Herbal, Integrative medicine, Research trends, Traditional Chinese medicine, Traditional medicine

## Abstract

**Background:**

Coronavirus disease 2019 (COVID-19) is a novel infectious disease caused by severe acute respiratory syndrome coronavirus 2, and responsible for a global pandemic. Despite there being no known vaccines or medicines that prevent or cure COVID-19, many traditional, integrative, complementary and alternative medicines (TICAMs) have been touted as the solution, as well as researched as a potential remedy globally. This study presents a bibliometric analysis of global research trends at the intersection of TICAM and COVID-19.

**Methods:**

SCOPUS, MEDLINE, EMBASE, AMED and PSYCINFO databases were searched on July 5, 2020, with results being exported on the same day. All publication types were included, however, articles were only deemed eligible if they made mention of one or more TICAMs for the potential prevention, treatment, and/or management of COVID-19 or a health issue indirectly resulting from the COVID-19 pandemic. The following eligible article characteristics were extracted: title; author names, affiliations, and countries; DOI; publication language; publication type; publication year; journal (and whether it is TICAM-focused); 2019 impact factor, and TICAMs mentioned.

**Results:**

A total of 296 eligible articles were published by 1373 unique authors at 977 affiliations across 56 countries. The most common countries associated with author affiliation included China, the United States, India and Italy. The vast majority of articles were published in English, followed by Chinese. Eligible articles were published across 157 journals, of which 33 were TICAM-focused; a total of 120 journals had a 2019 impact factor, which ranged from 0.17 to 60.392. A total of 327 TICAMs were mentioned across eligible articles, with the most common ones including: traditional Chinese medicine (*n* = 94), vitamin D (*n* = 67), melatonin (*n* = 16), phytochemicals (*n* = 12), and general herbal medicine (*n* = 11).

**Conclusions:**

This study provides researchers and clinicians with a greater knowledge of the characteristics of articles that been published globally at the intersection of COVID-19 and TICAM to date. At a time where safe and effective vaccines and medicines for the prevention and treatment of COVID-19 have yet to be discovered, this study provides a current snapshot of the quantity and characteristics of articles written at the intersection of TICAM therapies and COVID-19.

**Supplementary Information:**

The online version contains supplementary material available at 10.1186/s12906-020-03151-8.

## Background

The novel coronavirus disease 2019 (COVID-19) is an infectious disease caused by severe acute respiratory syndrome coronavirus 2 (SARS-CoV-2). Originating from Wuhan, China, it was first identified in December 2019, and has resulted in an ongoing global pandemic [1]. As of October 24th, 2020, more than 42 million cases have been reported across 218 countries and territories, resulting in more than 1.1 million deaths [2]. While certain practices such as physical distancing, self-isolation and frequent handwashing mitigate the spread of COVID-19, to date there are no vaccines or medications have been shown to prevent or treat the disease [1]. Despite this, a wide plethora of traditional, integrative, complementary and alternative medicines (TICAMs) have been touted as the solution, despite the paucity of evidence surrounding the safety and effectiveness of such therapies [3–5]. National governments have taken a wide-range of stances on TICAMs; China and India are among those promoting their respective traditional medicines [6–8]. In contrast, government mention of TICAMs in the context of COVID-19 typically come in the form of warnings of potential harm and fraudulent claims in Western countries such as the United States, Canada and Australia [5, 9, 10].

Despite varying government stances, it can be argued that all governments globally have a vested interest in researching promising COVID-19 therapies, which undoubtedly includes TICAMs. A number of initiatives have been launched to support ongoing research in this area, including the establishment of the Traditional, Complementary and Integrative Health and Medicine COVID-19 Support Registry [11]. Additionally, in early May 2020, the World Health Organization’s Regional Office for Africa issued a statement of support for scientifically-proven traditional medicine in the search for potential treatments for COVID-19 [12]. Unsurprisingly, there has been an uptick in the amount of research being conducted at the intersection of TICAM and COVID-19 and preliminary searches on academic databases such as PubMed or Google Scholar indicate a growing number of peer-reviewed publications.

To date, no study has assessed the characteristics of these publications, thus the purpose of the present study is to conduct a bibliometric analysis of global research trends at the intersection of TICAM and COVID-19. This bibliometric analysis provides current insight into the most commonly researched TICAM therapies, the institutions leading the studies, and the journals publishing the findings. Thus, this study’s findings are relevant to researchers and practitioners internationally, as it summarizes early and emerging research and may aid to provide a current snapshot of the quantity and characteristics of articles written at the intersection of TICAM therapies and COVID-19.

## Methods

Searches were conducted on SCOPUS, MEDLINE, EMBASE, AMED and PSYCINFO databases on July 5, 2020. Searches were all conducted with search results exported on the same day to prevent discrepancies between daily database updates. While recently published COVID-19-related bibliometric analyses have commonly employed searches across only one or two databases [13–15], multiple databases were selected in the present study to maximize the quantity of peer-reviewed publications captured at the intersection of TICAM and COVID-19. As TICAM is comprised of a very wide-range of therapies, the development of a comprehensive search strategy was informed by both a comprehensive list of TICAM therapies provided by the National Center for Complementary and Integrative Health [16], as well as a past textual analysis of common terms used to describe TICAM conducted by the author [17]. Only articles published in 2020 were included, given that the emergence of COVID-19 is very novel. In addition to a comprehensive search strategy, all articles were manually screened for by title and abstract for eligibility, by JYN and another research assistant; articles were only included if they made mention of one or more TICAMs for the potential prevention, treatment, and/or management of COVID-19 or a health issue indirectly resulting from the COVID-19 pandemic. Articles containing mention of TICAM, but not COVID-19, or vice versa were excluded. No restrictions were placed on article type or language. The complete SCOPUS and OVID (latter aforementioned four databases) search strategies are provided in Appendix 1.

The following data were extracted from each eligible article: title, author names, affiliations, author affiliated countries, DOI, language of publication, publication type, year of publication, journal, whether the article was published in a TICAM-focused journal, 2019 journal impact factor, and type(s) of TICAM mentioned. The impact factor of the journals as reported by InCites Journal Citation Reports was used [18]. Global trends associated with all eligible articles were identified and presented.

## Results

**Fig. 1:**
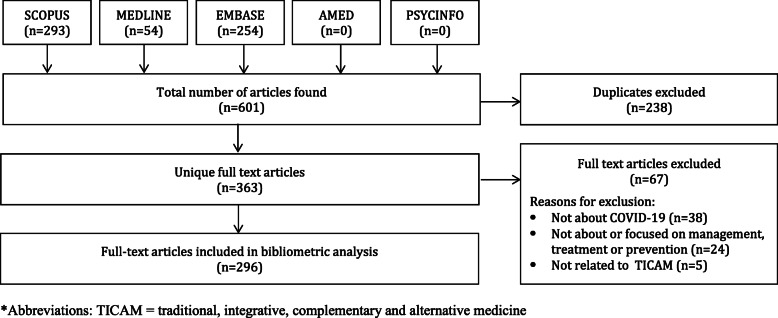
Bibliometric Analysis Flowchart. *Abbreviations: TICAM = traditional, integrative, complementary and alternative medicine

Searches across all academic databases retrieved a total of 601 titles, of which 238 were duplicate and 363 were unique. Of the unique articles, 296 were eligible as they mentioned at least one TICAM for the potential management, treatment and/or prevention of COVID-19 or COVID-19-induced conditions. The remaining 67 full-text articles were excluded for the following reasons: not about COVID-19 (*n* = 38); not about or focused on management, treatment, prevention of COVID-19 or COVID-19 induced conditions (*n* = 24); and not related to TICAM (*n* = 5). A bibliometric analysis flowchart is provided in Fig. 1.

Across all 296 publications and 1556 authors, 1373 were unique. The number of publications per author ranged from 1 to 7. Authors were affiliated with a total of 977 affiliations across 56 countries. Authors were all from the same affiliation country in 222 publications, two countries in 42 publications, three countries in 17 publications, four countries in 6 publications and two single publications contained 8 and 11 countries of affiliation. The number of articles containing affiliations from the following countries were as follows: China (*n* = 105), United States (*n* = 51), India (*n* = 33), Italy (*n* = 26), England (*n* = 24), Ireland (*n* = 12), Australia (*n* = 11), Canada (*n* = 9), Spain (n = 9), Turkey (*n* = 8), Hong Kong (*n* = 7), Iran (*n* = 6), Pakistan (*n* = 6), Saudi Arabia (*n* = 6), Brazil (*n* = 5), Egypt (n = 5), Germany (*n* = 5), South Korea (*n* = 5), Switzerland (*n* = 5) and Vietnam (*n* = 4). Additionally, three affiliations each were associated with the following countries: Bangladesh, Belgium, Denmark, France, Malaysia, Romania, Singapore, and Thailand. Two affiliations each was associated with the following countries: Algeria, Argentina, Austria, Greece, Jordan, Portugal, Scotland, Sweden, and Taiwan. One affiliation each was associated with the following countries: Chile, Colombia, Croatia, Estonia, Finland, Hungary, Indonesia, Israel, Japan, Lebanon, Netherlands, New Zealand, Nigeria, Poland, Russia, Slovakia, South Africa and Wales. Seven articles did not have declared affiliations/countries associated with their articles. Eligible articles were primarily published English (*n* = 251), followed by Chinese (*n* = 35), German (*n* = 4), and Italian (*n* = 2). Additionally, four of the articles published in English were also published in an additional language, Spanish. Eligible articles found were indexed by the academic databases searched as the following publication types: article (*n* = 166), letter (*n* = 62), review (*n* = 47), editorial (*n* = 12), note (*n* = 6), erratum (*n* = 1), preprint (*n* = 1) and short survey (*n* = 1). The general characteristics of eligible articles are summarized in Table 1. In addition, articles that had received at least 10 citations as of the search date in the SCOPUS database are provided in Table 2.

**Table 1:** General Characteristics of Eligible Articles

Number of Publications	296
Number of Authors	1556 (1373 unique)
Number of Publications Per Author	1 (*n* = 1246)
	2 (*n* = 93)
	3 (*n* = 20)
	4 (*n* = 9)
	5 (*n* = 3)
	6 (*n* = 1)
	7 (*n* = 1)
Number of Countries Affiliated Per Publication	1 (*n* = 222)
	2 (*n* = 42)
	3 (*n* = 17)
	3+ (*n* = 8)
Most Commonly Affiliated Countries	China (*n* = 105)
	United States (*n* = 51)
	India (*n* = 33)
	Italy (*n* = 26)
Language of Publication	English (*n* = 251)
	Chinese (*n* = 35)
	German (*n* = 4)
	Italian (*n* = 2)
Publication Types	Article (*n* = 166)
	Letter (*n* = 62)
	Review (*n* = 47)
	Editorial (*n* = 12)
	Note (*n* = 6)
	Erratum (*n* = 1)
	Preprint (*n* = 1)
	Short survey (*n* = 1)

**Table 2:** Most Highly Cited Articles (≥10 Citations)^a^

Article Title	Authors	Number of Citations
Evidence that vitamin d supplementation could reduce risk of influenza and covid-19 infections and deaths	Grant W.B., Lahore H., McDonnell S.L., Baggerly C.A., French C.B., Aliano J.L., Bhattoa H.P.	65
Traditional Chinese medicine for COVID-19 treatment	Ren J.-L., Zhang A.-H., Wang X.-J.	35
Traditional Chinese medicine in the treatment of patients infected with 2019-new coronavirus (SARS-CoV-2): A review and perspective	Yang Y., Islam M.S., Wang J., Li Y., Chen X.	32
In silico screening of Chinese herbal medicines with the potential to directly inhibit 2019 novel coronavirus	Zhang D.-H., Wu K.-L., Zhang X., Deng S.-Q., Peng B.	31
Lianhuaqingwen exerts anti-viral and anti-inflammatory activity against novel coronavirus (SARS-CoV-2)	Runfeng L., Yunlong H., Jicheng H., Weiqi P., Qinhai M., Yongxia S., Chufang L., Jin Z., Zhenhua J., Haiming J., Kui Z., Shuxiang H., Jun D., Xiaobo L., Xiaotao H., Lin W., Nanshan Z., Zifeng Y.	30
COVID-19: Melatonin as a potential adjuvant treatment	Zhang R., Wang X., Ni L., Di X., Ma B., Niu S., Liu C., Reiter R.J.	22
A molecular modeling approach to identify effective antiviral phytochemicals against the main protease of SARS-CoV-2	Islam R., Parves M.R., Paul A.S., Uddin N., Rahman M.S., Mamun A.A., Hossain M.N., Ali M.A., Halim M.A.	17
Stilbene-based natural compounds as promising drug candidates against COVID-19	Wahedi H.M., Ahmad S., Abbasi S.W.	16
Letter: Covid-19, and vitamin D	Panarese A., Shahini E.	12
Optimisation of vitamin d status for enhanced immuno-protection against covid-19	McCartney D.M., Byrne D.G.	11
Editorial: low population mortality from COVID-19 in countries south of latitude 35 degrees North supports vitamin D as a factor determining severity	Rhodes J.M., Subramanian S., Laird E., Kenny R.A.	10
A new clinical trial to test high-dose vitamin C in patients with COVID-19	Carr A.C.	10
Traditional Chinese medicine is a resource for drug discovery against 2019 novel coronavirus (SARS-CoV-2)	Ling C.-Q.	10

^a^Based on SCOPUS data only

In total, the 296 eligible articles were published in a total of 157 journals, of which 33 were identified to be TICAM-focused journals. Ninety articles were published in TICAM-focused journals. After hand-searching each journal on InCites Journal Citation Reports, it was found that 120 journals had a 2019 impact factor. Of these 120 journals, impact factors ranged widely from 0.17 to 60.392. In total, 193 articles were published in a journal with a 2019 impact factor. The number of articles published per journal ranged from 1 to 15; details about the fifteen journals with the highest number of articles are provided in Table 3.

**Table 3:** Top 14 Most Published Journals

Journal	TICAM Journal	2019 Journal Impact Factor	Number of Articles
Chinese Traditional and Herbal Drugs	Yes	N/A	15
Journal of Biomolecular Structure & Dynamics	No	N/A	14
Zhongguo Zhongyao Zazhi (China Journal of Chinese Materia Medica)	Yes	N/A	13
Pharmacological Research	No	5.893	12
Alimentary Pharmacology and Therapeutics	No	7.515	8
Irish Medical Journal	No	N/A	7
Life Sciences	No	3.647	6
Nutrients	No	4.546	6
Journal of Alternative & Complementary Medicine	Yes	2.109	5
World Journal of Traditional Chinese Medicine	Yes	N/A	5
Chiropractic and Manual Therapies	Yes	1.512	4
Diabetes and Metabolic Syndrome: Clinical Research and Reviews	No	N/A	4
Journal of Integrative Medicine	Yes	2.446	4
Medicine	No	1.552	4
Medicine in Drug Discovery	No	N/A	4

**Fig. 2:**
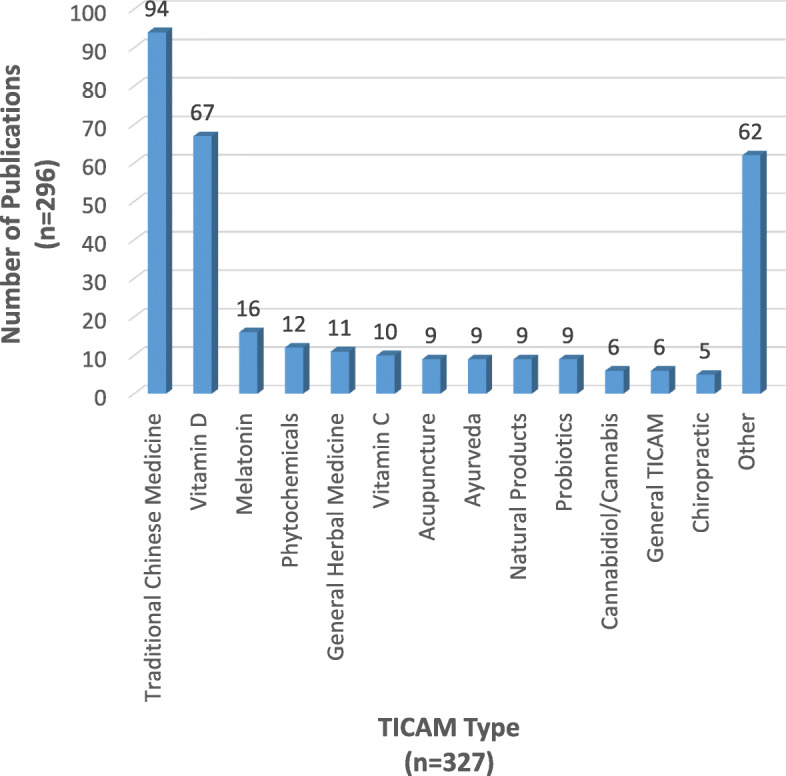
Most Common TICAMs across Eligible Publications

A total of 327 TICAMs (60 unique) were mentioned across the 296 eligible articles, as follows: traditional Chinese medicine (*n* = 92), vitamin D (*n* = 63), melatonin (*n* = 15), phytochemicals (*n* = 10), general herbal medicine (*n* = 9), vitamin C (*n* = 9), Ayurveda (*n* = 8), natural products (*n* = 8), probiotics (*n* = 7), cannabidiol/cannabis (n = 6), chiropractic (*n* = 5), general TICAM (*n* = 5), acupuncture (*n* = 3), homeopathy (*n* = 3), marine natural products (*n* = 3), antioxidants (*n* = 2), flavonoids (*n* = 2), tea (*n* = 2), celastrol (*n* = 1), dietary supplements (*n* = 1), fungi (*n* = 1), garlic (*n* = 1), garlic essential oil (*n* = 1), glycyrrhetinic acid (*n* = 1), hispidin, lepidine E and folic acid (*n* = 1), indigenous herbal medicine (*n* = 1), marine algal antioxidants (*n* = 1), massage (*n* = 1), meditation/mindfulness (*n* = 1), microbial natural products (*n* = 1), opioids and cannabinoids (*n* = 1), phytotherapy (*n* = 1), silvestrol (*n* = 1), spinal manipulative therapy (*n* = 1), stilbenes (*n =* 1), traditional Mongolian medicine (*n* = 1), traditional Persian medicine (*n* = 1), turmeric (*n* = 1), vitamin K antagonists (*n* = 1), vitamins and trace elements (*n* = 1), yoga (*n* = 1) and zinc iodide and dimethyl sulfoxide (*n* = 1). A number of studies incorporated one or more other TICAMs in combination with the aforementioned therapies as follows: moxibustion (*n* = 5, with acupuncture), ascorbic acid, zinc and N-acetylcysteine (*n* = 1, with vitamin d) curcumin and glycyrrhizic acid (*n* = 1, with vitamin c), essential oils (*n* = 1, with phytochemicals), essential oils and phytochemicals (*n* = 1, with general herbal medicine), exercise (*n* = 1, with vitamin d), guided relaxation (*n* = 1, with meditation), melatonin (*n* = 1, with vitamin d), natural products (*n* = 1, with general herbal medicine), plant bioactives (*n* = 1, with probiotics), probiotics and nutraceuticals (*n* = 1, with dietary supplements) quercetin and estradiol (n = 1, with vitamin d), traditional Chinese medicine (*n* = 1 with acupuncture; *n* = 1, with general TICAM) and yoga (*n* = 1, with Ayurveda; *n* = 1, with meditation). This is summarized in Fig. 2.

The entire dataset containing all of the aforementioned characteristics of all eligible articles is provided in Supplementary File 1 for the benefit of researchers and clinicians who seek to read the original publications, use this data to support further research, and foster future collaborations to investigate promising TICAMs in combatting the ongoing COVID-19 pandemic.

## Discussion

The present study provides a current and needed bibliometric analysis of global research trends at the intersection of COVID-19 and TICAM, as of July 2020. A wide-range of efforts are taking place globally to investigate TICAMs with the potential to prevent, treat and/or manage COVID-19, with the most research-productive countries being China, the United States, India and Italy. These findings are not entirely surprising, given that researchers in China likely initiated research earlier than the rest of the world as this is where the virus originated [1]. The United States and Italy are two countries that have suffered some of the largest casualties as a result of the global pandemic [2]. Like China, India also has a centuries-old traditional medicine system [19, 20], offering various TICAMs that could be potentially repurposed to treat COVID-19 patients [21]. It is also unsurprising that the vast majority of eligible articles were published in the English language, as this is considered the most widely-used language to disseminate research findings; additionally, the vast majority of articles indexed in the databases searched are written in English [22].

Eligible articles were found to have been published in a wide-range of journals, covering a wide-range of topics and disciplines. Only about one fifth of the total number of articles were published in a TICAM-focused journal. One particularly important finding is the fact that over 20% of eligible articles were not published in a journal with an InCites Journal Citation Reports impact factor as of 2019 [18]. Despite all eligible articles being indexed in either the SCOPUS or an OVID database, and while acknowledging that the impact factor is not without its limitations, nor is it the only metric by which to assess the true “impact” of research conducted [23, 24], this finding does, however, draws into question the potential quality and readership of such studies.

In terms of TICAMs mentioned, it was found that a disproportionately large number of articles mentioned traditional Chinese medicine, beyond the fact that the vast majority of these articles were published by researchers with an affiliation in China. Interestingly, almost all TICAMs mentioned across all articles were either natural health products (i.e. vitamins, herbs), or a system of traditional medicine that incorporates these supplements. Vitamin D, melatonin, and vitamin C were also relatively commonly discussed across eligible articles found. Surprisingly little research has been published at the intersection of COVID-19 and modalities such as mind-body medicine, manipulative and body-based practices, as well as TICAM whole medical systems outside of traditional Chinese Medicine and Ayurveda, such as naturopathy and chiropractic. Thus, it is hoped that this study can be of value internationally to researchers and clinicians with an interest in TICAM by offering a current snapshot of the current research being conducted and published at this intersection, including knowledge of what topics are being studied, by whom and where. Furthermore, TICAM experts may find Supplementary File 1 useful in quickly identifying research projects that they may not have otherwise known about, potentially leading to fruitful collaborations, especially at this crucial time where safe and effective vaccines and medicines for the prevention and treatment of COVID-19 have yet to be identified.

### Comparison to broader COVID-19 bibliometric analyses

While it appears that this is the first bibliometric analysis to be conducted at the intersection of COVID-19 and TICAM research, it is worth mentioning that a number of bibliometric analyses of COVID-19 research have been published over the recent months [25–30]. Some general comparisons can be drawn to the present study’s findings; for example, many of these bibliometric analyses identified that the greatest proportion of publications originated from China and the United States [25–28, 30]. Unsurprisingly, all studies all indicated a large uptick in the number of publications in the months following January 2020, as COVID-19 became a global research priority [25–30]. One study sought to compare the research between English and Chinese studies, finding that a greater number of keywords existed across English publications, and that traditional Chinese medicine was written about more frequently in Chinese studies [29]. While bibliometric analyses conducted at specific intersections (COVID-19 and TICAM) or with respect to more specialized aspects (i.e. language, such as the one published by Fan et al. [29]), provide a narrower view of publication landscapes, the advantages of such approaches include a more comprehensive understanding of the trends in research taking place and the needs identified specific to such intersections/aspects.

### Strengths and limitations

This bibliometric study contained a number of notable strengths including the fact that a highly comprehensive search comprised of a wide-range of TICAM-related search terms was developed. Another strength includes the fact that searches were conducted across five unique and large academic databases, thus capturing the vast majority of indexed literature that has been published at the intersection of COVID-19 and TICAM. While articles published in any language were included in the bibliometric analysis itself, one limitation includes the fact that no non-English academic databases were searched. Furthermore, even English-language articles published in reputable journals would not have been captured if they were not indexed in the five databases searched. Additionally, the number of citations per article were not provided as only the SCOPUS database provides this metric. This is a common reason why many bibliometric analyses only report searches on this single database, however, given that this literature is very new and much is likely to change in the coming months, it was decided that this was not the most relevant nor important metric to capture for the purpose of the present study. Lastly, and while not a methodological limitation, it is worth mentioning that the present bibliometric analysis only captures research published up to early July 2020; given that new COVID-19-specific research continues to be published each day, an update of this study is warranted as a future direction.

## Conclusions

The present study is the first bibliometric analysis to date of global research trends at the intersection of COVID-19 and TICAM. Findings include the fact that a wide-range of TICAMs have been mentioned across articles found; a total of 327 TICAMs were mentioned across the 296 eligible articles with the vast majority of them including traditional Chinese medicine and vitamin D supplementation. Eligible articles were published by a total of 1373 authors with affiliations in 56 countries, the most common of which included China, the United States, India and Italy. At a time where safe and effective vaccines and medicines for the prevention and treatment of COVID-19 have yet to be discovered, this study provides researchers and clinicians with a greater knowledge of the characteristics of articles that been published at the intersection of COVID-19 and TICAM to date.

### Supplementary Information

**Additional file 1.** Supplementary file 1. Characteristics of All Eligible Articles Published at the Intersection of COVID-19 and TICAM.

## Data Availability

All data generated or analysed during this study are included in this published article [and supplementary file].

## Appendix 1

**Table 4:** Search Strategies

**SCOPUS Search Strategy**	
Executed July 05, 2020 (TITLE((Acai) or (Acupuncture) or (“*Aloe Vera*”) or (“Alternative Medicine*”) or (“Alternative Therap*”) or (Antioxidants) or (Aromatherapy) or (“Asian Ginseng”) or (Astragalus) or (Ayurved*) or (Bilberry) or (“Bitter Orange”) or (“Black Cohosh”) or (Bromelain) or (Butterbur) or (Cannab*) or (“Cat’s Claw”) or (Chamomile) or (Chasteberry) or (“Chelation Therapy”) or (Chinese Patent Medicine*) or (Chiropract*) or (Chondroitin) or (Cinnamon) or (Cleanse*) or (“Coenzyme Q10”) or (“Colloidal Silver”) or (“Complementary and Alternative Medicine*”) or (“Complementary and Alternative Therap*”) or (“Complementary and Integrat*”) or (“Integrat* and Complementary”) or (“Complementary Medicine*”) or (“Complementary Therap*”) or (CoQ10) or (Cranberry) or (Cupping) or (Dandelion) or (Detox*) or (“Dietary Supplement*”) or (“Dimethyl sulfoxide”) or (Echinacea) or (“Energy Drink*”) or (Ephedra) or (“European Elder”) or (“European Mistletoe”) or (“Evening Primrose*”) or (Fenugreek) or (Feverfew) or (Flaxseed*) or (“Garcinia Cambogia”) or (Garlic) or (Ginger) or (Ginkgo) or (Glucosamine) or (Goldenseal) or (“Grape Seed Extract”) or (“Green Tea”) or (“Guided Imagery”) or (Hawthorn) or (Herb*) or (Homeopath*) or (Hoodia) or (“Horse Chestnut”) or (Hypnosis) or (“Integrat* Medicine*”) or (“Integrat* Therap*”) or (Kava) or (Kratom) or (Lavender) or (Lianhuaqingwen) or (“Lianhua qingwen”) or (“Licorice Root”) or (Magnet*) or (Marijuana) or (Massage) or (Meditation) or (Melatonin) or (Methylsulfonylmethane) or (“Milk Thistle”) or (“Mind-Body Medicine*”) or (“Mind-Body Therap*”) or (Mineral*) or (Natural Compound*) or (Natural Health Product*) or (“Natural Medicine*”) or (“Natural Product*) or (“Natural Therap*) or (Naturopath*) or (Noni) or (“Omega-3 Fatty Acid*”) or (Passionflower) or (“Peppermint Oil”) or (Phyto*) or (Pomegranate) or (Probiotic*) or (“Progressive Relaxation”) or (“Qi Gong”) or (“Red Clover”) or (“Red Yeast Rice”) or (Reflexology) or (Reiki) or (“Relaxation Technique*”) or (Rhodiola) or (“S-Adenosyl-L-methionine”) or (Sage) or (“Saw Palmetto”) or (Soy) or (“Spinal Manipulation*”) or (“St. John’s Wort”) or (“Tai Chi”) or (TCM) or (“Tea Tree Oil”) or (Tea) or (Thunder God Vine) or (“Traditional Medicine*”) or (“Traditional Therap*”) or (“Traditional Chinese Medicine*”) or (Turmeric) or (Valerian) or (Vitamin*) or (Yoga) or (Yohimbe))) AND ((covid-19) OR (covid19) or (coronavirus) or (“Coronavirus Disease 2019″) or (“Severe Acute Respiratory Syndrome Coronavirus 2″) or (“SARS-CoV-2″)) AND (LIMIT-TO (PUBYEAR,2020))	
**OVID Search Strategy (MEDLINE, EMBASE, AMED, PSYCINFO)**	
Database: AMED (Allied and Complementary Medicine) < 1985 to June 2020>, Embase < 1974 to 2020 July 02>, APA PsycInfo < 1806 to June Week 52,020>, Ovid MEDLINE (R) and Epub Ahead of Print, In-Process & Other Non-Indexed Citations, Daily and Versions (R) < 1946 to July 02, 2020> Search Strategy: -------------------------------------------------------------------------------- 1 (Acai or Acupuncture or Aloe Vera or Alternative Medicine* or Alternative Therap* or Antioxidants or Aromatherapy or Asian Ginseng or Astragalus or Ayurved* or Bilberry or Bitter Orange or Black Cohosh or Bromelain or Butterbur or Cannab* or Cat’s Claw or Chamomile or Chasteberry or Chelation Therapy or Chinese Patent Medicine* or Chiropract* or Chondroitin or Cinnamon or Cleanse* or Coenzyme Q10 or Colloidal Silver or (Complementary and Alternative Medicine*) or (Complementary and Alternative Therap*) or (Complementary and Integrat*) or (Integrat* and Complementary) or Complementary Medicine* or Complementary Therap* or CoQ10 or Cranberry or Cupping or Dandelion or Detox* or Dietary Supplement* or Dimethyl sulfoxide or Echinacea or Energy Drink* or Ephedra or European Elder or European Mistletoe or Evening Primrose* or Fenugreek or Feverfew or Flaxseed* or Garcinia Cambogia or Garlic or Ginger or Ginkgo or Glucosamine or Goldenseal or Grape Seed Extract or Green Tea or Guided Imagery or Hawthorn or Herb* or Homeopath* or Hoodia or Horse Chestnut or Hypnosis or Integrat* Medicine* or Integrat* Therap* or Kava or Kratom or Lavender or Lianhuaqingwen or Lianhua qingwen or Licorice Root or Magnet* or Marijuana or Massage or Meditation or Melatonin or Methylsulfonylmethane or Milk Thistle or Mind-Body Medicine* or Mind-Body Therap* or Mineral* or Natural Compound* or Natural Health Product* or Natural Medicine* or Natural Product* or Natural Therap* or Naturopath* or Noni or Omega-3 Fatty Acid* or Passionflower or Peppermint Oil or Phyto* or Pomegranate or Probiotic* or Progressive Relaxation or Qi Gong or Red Clover or Red Yeast Rice or Reflexology or Reiki or Relaxation Technique* or Rhodiola or S-Adenosyl-L-methionine or Sage or Saw Palmetto or Soy or Spinal Manipulation* or “St. John’s Wort” or Tai Chi or TCM or Tea Tree Oil or Tea or Thunder God Vine or (Traditional adj3 (Medicine or Therap*)) or (Traditional Chinese adj3 Medicine*) or Turmeric or Valerian or Vitamin* or Yoga or Yohimbe).ti. (1314477) 2 (Coronavirus or COVID-19 or COVID19 or Coronavirus or “Coronavirus Disease 2019” or “Severe Acute Respiratory Syndrome Coronavirus 2” or “SARS-CoV-2”).mp. (87836) 3 1 and 2 (609) 4 remove duplicates from 3 (370) 5 limit 4 to yr = “2020 -Current” (308) ***************************	

## Abbreviations

COVID-19: Coronavirus disease 2019
TICAM: Traditional, integrative, complementary and alternative medicine

## References

[1] World Health Organization. Q&a on coronaviruses (COVID-19). 2020; Assessed 24 October 2020: https://www.who.int/emergencies/diseases/novel-coronavirus-2019/question-and-answers-hub/q-a-detail/q-a-coronaviruses.

[2] World Health Organization (2020). Coronavirus disease (COVID-19) pandemic assessed 24 October 2020.

[3] Liu M, Caputi TL, Dredze M, Kesselheim AS, Ayers JW (2020). Internet searches for unproven COVID-19 therapies in the United States. JAMA Intern Med.

[4] National Center for Complementary and Integrative Health (2020). In the news: coronavirus and “alternative” treatments.

[5] Food and Drug Administration (2020). Coronavirus update: FDA and FTC warn seven companies selling fraudulent products that claim to treat or prevent COVID-19.

[6] National Health Commission of the People’s Republic of China. Diagnosis and Treatment Protocol for COVID-19 (Trial Version 7). Assessed 07 June 07 2020: http://en.nhc.gov.cn/2020-03/29/c_78469.htm.

[7] Cyranoski D. China is promoting coronavirus treatments based on unproven traditional medicines. Nature. 2020. 10.1038/d41586-020-01284-x. 10.1038/d41586-020-01284-x 32376938

[8] Ministry of AYUSH. Advisory for corona virus— homoeopathy for prevention of corona virus infections, Unani medicines useful in symptomatic management of corona virus infection. Online document at Assessed 07 June 07 2020: https://pib.gov.in/PressReleasePage.aspx?PRID=1600895.

[9] Government of Canada (2020). Health products that make false or misleading claims to prevent, treat or cure COVID-19 may put your health at risk.

[10] Australian Government Department of Health Therapeutic Goods Administration (2020). Warning about products claiming to treat or prevent the novel coronavirus.

[11] Helfgott Research Institute. Traditional, Complementary and Integrative Health and Medicine COVID-19 Support Registry. Online document at Assessed 07 June 07 2020: https://redcap.nunm.edu/redcap/surveys/?s=PE3EHAYDT3.

[12] World Health Organization. Regional Office for Africa. (2020c) WHO supports scientifically-proven traditional medicine. Assessed 07 June 07 2020: https://www.afro.who.int/news/who-supports-scientifically-proven-traditional-medicine.

[13] Radanliev P, De Roure D, Walton R, Van Kleek M, Santos O, Montalvo RM (2005). What country, university or research institute, performed the best on COVID-19? Bibliometric analysis of scientific literature. EPMA J.

[14] Chahrour M, Assi S, Bejjani M, Nasrallah AA, Salhab H, Fares M (2020). A bibliometric analysis of Covid-19 research activity: a call for increased output. Cureus.

[15] Hossain MM. Current status of global research on novel coronavirus disease (Covid-19): A bibliometric analysis and knowledge mapping. Hossain MM. Current status of global research on novel coronavirus disease (COVID-19): a bibliometric analysis and knowledge mapping [version 1. 2020 May 18.

[16] National Center for Complementary and Integrative Health (2020). Health Topics A–Z. Assessed 07 June 07 2020: https://www.nccih.nih.gov/health/atoz.

[17] Ng JY, Boon HS, Thompson AK, Whitehead CR (2016). Making sense of “alternative”, “complementary”, “unconventional” and “integrative” medicine: exploring the terms and meanings through a textual analysis. BMC Complement Altern Med.

[18] Clarivate Analytics (2020). Cites journal citation reports.

[19] National Center for Complementary and Integrative Health (2020). Traditional Chinese medicine: what you need to know.

[20] National Center for Complementary and Integrative Health (2020). Ayurvedic medicine. Depth.

[21] Vellingiri B, Jayaramayya K, Iyer M, Narayanasamy A, Govindasamy V, Giridharan B (2020). COVID-19: a promising cure for the global panic. Sci Total Environ.

[22] Ammon U (2011). The dominance of English as a language of science: effects on other languages and language communities. Walter de Gruyter.

[23] Lariviere V, Kiermer V, MacCallum CJ, McNutt M, Patterson M, Pulverer B (2016). A simple proposal for the publication of journal citation distributions. BioRxiv..

[24] Index N (2018). What’s wrong with the journal impact factor in 5 graphs.

[25] Gong Y, Ma TC, Xu YY, Yang R, Gao LJ, Wu SH (2020). Early research on COVID-19: a bibliometric analysis. Innovation.

[26] Yang F, Zhang BS, Wang Q, Zhang Q, Han J, Wang L, Qing, Zhang, Qi, Han, Junming, Wang, Lijie, Wu, Xinying MM, Pan, Fengming MM, Xue F (2020). Analysis of the global situation of COVID-19 research based on Bibliometrics. Analysis of the global situation of COVID-19 research based on Bibliometrics (5/8/2020).

[27] Sa’ed HZ, Al-Jabi SW (2020). Mapping the situation of research on coronavirus disease-19 (COVID-19): a preliminary bibliometric analysis during the early stage of the outbreak. BMC Infect Dis.

[28] Zyoud SH, Zyoud AH (2020). Coronavirus disease-19 in environmental fields: a bibliometric and visualization mapping analysis. Environ Dev Sustain.

[29] Fan J, Gao Y, Zhao N, Dai R, Zhang H, Feng X (2020). Bibliometric analysis on COVID-19: a comparison of research between English and Chinese studies. Front Public Health.

[30] Martinez-Perez C, Alvarez-Peregrina C, Villa-Collar C, Sánchez-Tena MÁ (2020). Citation network analysis of the novel coronavirus disease 2019 (COVID-19). Int J Environ Res Public Health.

